# Prevalence and associated factors of stunting and thinness among adolescent Somalian refugee girls living in eastern Somali refugee camps, Somali regional state, Southeast Ethiopia

**DOI:** 10.1186/s13031-019-0203-3

**Published:** 2019-05-17

**Authors:** Melaku Tadege Engidaw, Alemayehu Digssie Gebremariam

**Affiliations:** Public Health Department, College of Health Sciences, Debre Tabor University, P.o.box: 031 Debre Tabor, Ethiopia

**Keywords:** Refugee, Adolescent girls, Stunting, Thinness, And Somalia

## Abstract

**Background:**

Adolescence is a critical time of life stage with a continuum of physical, cognitive, behavioral and psychosocial changes. It is also a period of physical growth, reproductive maturation and cognitive transformations with the highest nutrient requirements. Among the macronutrient deficiencies, stunting and thinness are the most common nutritional problems in many parts of the world but the highest burden is in developing countries with the highest number of adolescents and the displaced population. Overall, there is a scarcity of studies on refugee adolescent girls stunting, thinness, and contributing factors. Therefore, this study aimed to fill this identified gap.

**Method:**

Cross-sectional study design was employed. A total of 423 adolescent refugee girls were selected by using simple random sampling technique. A structured & pre-tested questionnaire was used after translating into the Somali language to collect the data. The physical measurement of the height and the weight were done as per the standard. Descriptive statistics were employed. Variables were considered for multivariable logistic regression if a *P*-value was ≤0.2 during univariate logistic regression. The odds ratio with a 95% CI was calculated and a P-value of ≤0.05 was considered to declare the statistical significance of variables after fitting into the multivariable logistic regression.

**Result:**

A total of 415 adolescent girls was included in the study with a response rate of 98.1%. The overall prevalence of stunting and thinness was 9.7% (95% CI: 7.0, 12.3), and 15.2% (95% CI: 11.8, 18.9) respectively. The older adolescent girls were 2 (AOR: 2.10, 95% CI: 1.12, 3.93) times more likely to develop stunting as compared to younger adolescents. The pre-menarcheal adolescent girls were 64% (AOR: 0.36, 95% CI: 0.12, 0.75) less likely to be thin as compared to post-menarche.

**Conclusion:**

The prevalence of stunting and thinness among adolescent refugee girls was a low and moderate public health problem respectively. The stunting was significantly associated with the age and thinness was associated with the menarcheal status of the adolescent girls. So, stakeholders should enable intervention to encourage and increase the intake of calorie-dense food adapted to adolescent’s girl’s age and menarcheal status.

## Background

Adolescence is the period between 10 and 19 years of age which again classified as younger (10 - 14 yrs) and older (15 - 19 yrs) adolescents [1]. It is a continuum of physical, cognitive, behavioral and psychosocial change that is characterized by increasing levels of individual autonomy, a growing sense of identity, self-esteem and progressive independence from adults [2, 3]. It is a period of physical growth, reproductive maturation, and cognitive transformations in the life cycle which lead to the high requirement of macro or micronutrient or both [3–5]. The number of young people is increasing in the world and nearly half of the population in developing countries is under the age of 19 years [6]. In 2012, 1.2 billion adolescents were in this world and among them, 90% were found in developing countries with low and middle income which have a social, economic and political impact [7].

Macro and micronutrient deficiencies are due to inadequate food intake, poor nutrient content of the food and repeated infections. This problem is critical in developing countries where a majority of adolescents and displaced population is located [8–10].

Among macronutrient deficiencies, protein-energy malnutrition is one of the most common nutritional problems. The cause of protein-energy malnutrition is multifactorial but the underlining causes among children and women are the inadequate intake of foods and illness which is common among displaced and refugee community [11–14].

The deficiency of micro and/or micronutrient is very common in refugee settings since the major food sources are general ration. Due to this, women and children are vulnerable to stunting and wasting due to diseases burden, inadequate intake of nutrients and micronutrient deficiency [15–18]. The nutritional status of some refugees might be compromised because of dislocation/displacement, lack of income, and limited access to nutritious foods [15]. In Kakuma and Nepal refugee camps (in Kenya and Nepal respectively), adolescents with low BMI was 57.47 and 36.80% respectively [19]. Also, a high prevalence of low body mass index (BMI) was observed [20] which suggest the existence of wasting and stunting. Results from the first round of data collection (at the beginning of camp establishment) Sarajevo, Bosnia and Herzegovina showed a higher level of undernutrition among refugees (15.0, 95% CI (9.95, 20.05%)) as compared to residents (5.3, 95% CI (1.93, 8.67%)). Later, higher levels of undernutrition occurred among residents (8.1, 95% CI (4, 12.2%)) as compared to the refugees (7.0, 95% CI (3.4, 10.6%)) [21].

The prevalence of BMI, anemia, low vitamin A status, and signs of micronutrient deficiencies among adolescent refugees was common among the Nepalese refugee even during a fortified food distribution in the period of in 1999 due to micro or macronutrients or both [20].

The study among North Korean refugee boys and girls showed that the greatest gap observed in mid-teen years was caused by differences in growth tempos during the period of pubertal growth. The mean of weight for age Z score (WAZ) of the North Korean refugee adolescents was higher than their height for age Z score (HAZ), indicating that their growth in height is poorer than that of weight. The mean HAZ of North Korean refugee adolescents born later was not statistically significantly lower than that of those born earlier or during the famine. Those who stay in the refugee camp showed a positive effect on their growth status and suggesting that they experienced some degree of catch-up growth while they were staying in transit countries. Among all, sex, age at escape and measurement, the time interval between escape from North Korea and arrival in South Korea and year of escape were found to be significant factors in their growth status [22]. So, conducting this study is therefore very important as there are limited studies and knowledge on stunting and thinness and their associated factors among adolescent refugee girls.

## Methods and materials

### Study settings

The Aw-Barre refugee camp is found under Eastern Somalia refugee camp coordination office in Liben Zone of the regional state. It is located 678 km away from Addis Ababa, the capital city of Ethiopia, 78 km from Jigjiga, the regional capital of the Somali regional state of Ethiopia, and 7 km at the border of Somalia. It was established in July 2007 by United Nation Higher Commission for Refugee (UNHCR) and Administration of Refugee and Returnee Affairs (ARRA) of Ethiopia. The camp is located at an altitude of 1621.84 m (5321 ft’s) above sea level [23]. According to 2014 as of November ARRA report, the refugee camp had a total population of 12,803 and among this 5500 and 7382 were male and female, respectively. There are a number of different clans or ethnic groups, the majority of them were Hawuyie, Barob, Shekhal, Bantu, Asharaf, and others. Aw-Barre is one of the Eastern refugee camps in the Eastern part of Ethiopia for Somalia refugees. According to recent numbers from the camp data base report, there was a total of 1318 adolescent refugee girls aged 10–19 years in this refugee camp.

### Study design and period

Cross-sectional study design was employed from February to March 2015.

### Source and study population

The source population of the study was all the adolescent girls in Somali refugee camps, and the study population was adolescent girls in Aw-Barre refugee camp.

### Inclusion and exclusion criteria

All adolescent girls aged 10–19 years in Aw-Barre refugee camp were included in this study. Pregnant and lactating adolescent, adolescent with a physical disability and asylum seekers were excluded from this study.

### Sample size

The sample size was calculated using a single population proportion formula. During calculation, we were considered 95% of confidence level, 50% of the proportion of Stunting and/or Thinness, and 5% of marginal error. Then, the final sample size for this study was 423 after adding 10% of the nonresponse rate.

### Sampling procedures

Among the three refugees’ camps (Aw-Barre, Sheder, and Kebirebeya), Aw-Barre was selected by using the lottery method. In this camp, the total adolescent girls were 1318. A simple random sampling technique was applied to obtain the required sample size after gaining the lists of updated or revalidated adolescent refugee girls list with the respective house and block number from Aw – Barre refugee and returnee affairs bureau, refugee camp database manager office to use as a sampling frame. After the random selection, the data were collected from house to house.

### Data collection methods and equipment

Modified and pretested UNHCR standardized expanded nutrition survey (SENS) questionnaire was used to collect the data by interviewing the adolescent girls with their mother or caregiver. The questioner was predesigned and semi-structured. The parent’s interview was taken whenever to obtain relevant information regarding the socio-demographic characteristics, health, and diseases condition, dietary pattern, and physical measurements. The questionnaire had both open and close-ended questions. The English version of the questionnaire was translated into the local language (Somali) and was translated back into the English language to check its consistency by another skilled person.

A three days training with pretest was given for the data collectors and two field supervisors on the basic skills of hemoglobin measurement, calibration of instruments, interview techniques, obtaining the written consent or assent, and precautions during the data collection time. In total, there were 2 teams comprised of a supervisor, a medical laboratory and three nurses. During training, data collectors practiced weight and height measurements on each other. Before starting data collection, always there was checking of materials and equipment. The weights and heights were measured three times by using a weight scale with height stand machine at the Frankfurt position, and then the result was recorded to the nearest 0.1 kg and 0.1 cm respectively. Then the average of the three measurements of height and weight was taken. First, the finger was wiped with alcohol-soaked cotton, and then finger pricking was done. The pricked finger was gently pressed to get a sample of 10 μl blood on the HemoCuvettes and the HemoCuvetts was inserted into the HemoCueHb 301 machine. Finally, the hemoglobin level was read from the HemoCueHb 301 machine and recorded on the questionnaire. During the data collection time, communication between the data collectors, supervisors, and the principal investigators were held on a daily basis to update data collection progress.

### Data quality assurance

The pre-test was done outside of the study area (at Sheder refugees’ camp) on 20 samples (adolescent refugee girls) before the actual data collection period. Based on the pretest, corrective measures were done on the questionnaire. The weight scales were calibrated using 1 kg standard weight and the height measurements were checked with other meter taps. The HemoCueHb 301 machine was calibrated by using the 3 calibrating Hemo – solutions (EurotolHb 301 control solution which was a bovine-based solution). The pricing and taking of the sampled blood were taken after drying a weep finger by avoiding squeezing of the finger to avoiding air bubbles during filling of HemoCuvettes.

The definition of concepts and terms had been done clearly with the Somali language to avoid ambiguity. The supervisors and data collectors were recruited outside of the refugee health center to avoid information bias due to familiarization.

### Definitions of terms

**Poor nutritional status:** when the BMI of the adolescent girl was < 18.5 kg/m^2^ which is classified as severe, moderate and mild if the BMI ≤ 16 kg/m^2^, 16 – 17 kg/m^2^ and 17–18.4 kg/m^2^ respectively [24].

**Stunting:** when the adolescent girl height for age Z score was below -2SD from the median value of WHO reference data [25].

**Thinness/wasting:** when the adolescent girl BMI-for-age Z score was below −2SD from the median value of WHO reference data [25].

**Anemia among adolescents:** when the adjusted hemoglobin level was below 12.5 mg/dl [26] since the refugee camp is located 1000 m above sea level (+ 0.5 g/dl) [27].

**Dietary Diversity Score:** was the list of foods consumed within the last 24 h and was categorized as low (≤ 3 food groups), medium (4 and 5 food groups) and high dietary diversity score (≥ 6 food groups) [28]**.**

### Data processing and analysis

The collected data were checked for completeness and consistency by the supervisors and the principal investigator during and after the data collection period. The data were managed by editing, verification, coding, classification, and tabulation during data entry and analysis. The data were entered into Epi info 7 and WHO AnthroPlus software. The AnthroPlus software was used to calculate the Z score of HAZ and BMI for age. Finally, during data analysis, four flagged cases of HAZ (when the Z score is less than − 6 and above + 6) and WAZ (when the Z score is less than − 5 and above + 5) were excluded from this study. After this, all the data were transported into SPSS version 20 statistical software for the descriptive and analytical analysis. A univariate logistic regression model was used to assess the independent effects of each independent variable towards the development of adolescent refugee girls stunting and thinness.

Variables with a *p*-value ≤0.2 during a univariate analysis were fitted into a multivariable logistic regression model to identify the independent effect of each variable. After doing a multivariable logistic regression, a variable having a p-value of less than or equal to 0.05 was considered as a statistically significant. The odds ratio with a 95% confidence interval was used to assess the independent and multivariable effect. The Hosmer-Lemeshow goodness of fit test was performed and the *P*-value for stunting and thinness was 0.80 and 0.92 respectively.

## Result

### Socio-demography

Four hundred twenty-three refugee adolescent girls were selected randomly with a response rate of 415(98.10%). The mean ± SD of age was 13.94 + 2.74 years. From all, 244(58.79%) were found between the ages of 10 - 14 yrs. Only 2(0.50%) of them were involved in some activities to generate additional income. A quarter of adolescent was married (23(5.5%)) and three quarters (305(73.5%)) went to primary school shown in the table below (Table 1).

**Table 1:** Socio-demographic characteristics of adolescent refugee girls in Aw Barre Refugee Camp, Somali Regional state, Southeast Ethiopia, 2015. (*N* = 415)

Variables	Categories	Number	Percentage (%)
Age	10–14	244	58.79
	15–19	171	41.21
Marital status	Single	392	94.50
	Married	23	5.50
Ethnicity	Hawuyie	103	24.80
	Asharafa	39	9.40
	Bantu	75	18.10
	Dir	48	11.60
	Darod	27	6.50
	^a^Other	123	29.6
Family size	1–4	28	6.70
	5–9	222	53.50
	10–19	165	39.80
Educational status	Unable to read & write	45	10.80
	Able to read and write	17	4.10
	Primary school (1–8)	305	73.50
	Secondary school (9–12) & above	48	11.60
Household Rearing Domestic animals	Yes	9	2.20
	No	406	97.80
Selling of food aid items^b^	Yes	410	98.80
	No	5	1.20

^a^Others: Gore, Gaboye/Maddagan, Areb, Shikal, Tumal, Samaran, Isak, Barbo, Rahawayan, Shanshi, Geladi, Moreshe, Jalele and Durukbo

^b^To by their staple diets like Macaroni, Spaghetti, and rice

### Dietary diversity

All of the respondents were using rice, spaghetti, and macaroni as a staple diet of the family. The major sources of foods were the general ration which is donated and distributed by the World Food Program (WFP) and ARRA respectively. Most of the households (410(98.8%)) sold donated food to buy their staple food, and the common sold item was Wheat. A majority of respondents had a good diversity score (283 (68.2%)) while a third had a medium or poor dietary diversity score (113 (27.2%) and 19 (4.6%) respectively.

### Diseases and abnormal menstruation

None of the respondents report any acute or known chronic diseases as well as the use of medical drugs. More than half of the respondents 238(57.3%) were started menstruation during the data collection period and from those, only 3(0.7%) of them were reporting the existence of abnormal menstruation.

### Nutritional status of refugee adolescent girls

The physical measurements of the weight, height, and hemoglobin level were used to determine the nutritional status and the anemic status of all the respondents. In this study, the mean ± Standard Deviation (SD) of weight, height, and Hemoglobin (Hgb) of respondents were 43.27 kg ± 11.12 kg, 152.15 cm ± 9.69 cm, and 13.46 g/dl ± 1.48 g/dl respectively.

The average ± SD of respondents Body Mass Index (BMI) were 18.46 ± 3.63 kg/m^2^. Based on WHO classification, 110(26.5%), 56(13.5%) and 67(16.1%) had severe, moderate and mild malnutrition with respective of their BMI. Also, the risk of overweight and obesity was 16(3.9%) and 4(1.0%) respectively. Based on the Hgb level, 57(13.7, 95% CI (10.8, 17.1)) were anemic which was a mild public health problem in this refugee camp.

**Fig. 1:**
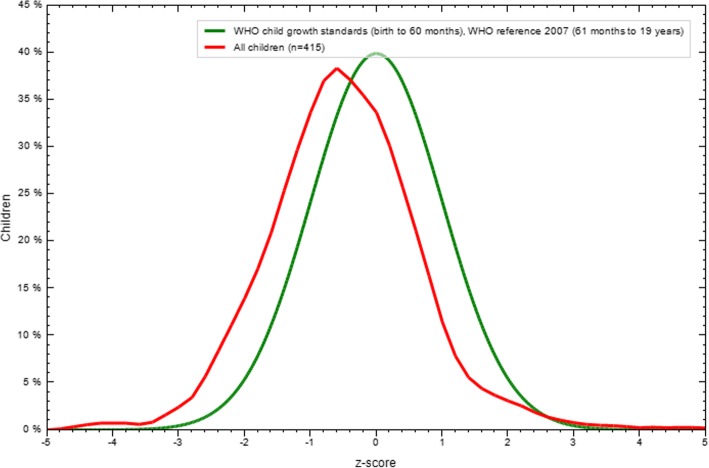
Height for age result for adolescent refugee girls in Aw – Barre Refugee Camp, Somali Regional state, Southeastern Ethiopia, 2015

One in 10 adolescent girls (9.7% (95% CI: 7.0, 12.3)) was stunted (HAZ < -2 z-score (WHO standard)) of which 1% severely stunted (HAZ < -3 z-score). The overall mean ± SD of HAZ z score was − 0.5 ± 1.13. From stunted adolescent girls, the rate of severely and moderately stunting was 1 and 8.7% respectively (Table 2). Older adolescents (15 - 19 yrs) (166) were more severely stunted (1.2%) as compared to younger adolescents (249) (10 - 14 yrs), 0.8%. The distribution of height for age Z score is illustrated in Fig. 1.

**Table 2:** Magnitude of stunting and thinness among adolescent refugee girls in the Aw-Barre Somalia refugee camp, Somali Regional state, Southeast Ethiopia, 2015

Age (yrs)	HFA with 95% CI				% of BMI for Age with 95% CI						
	< −3SD	< −2SD	Mean	SD	< −3SD	< −2SD	< +1SD	< +2SD	< +3SD	Mean	SD
10–14	0.8 (0, 2.1)	8.8 (5.1, 12.6)	−0.39	1.23	3.2 (0.8, 5.6)	16.5 (11.7, 21.3)	8.8 (5.1, 12.6)	2 (0.1, 4)	0 (0, 0.2)	−0.69	1.26
15–19	1.2 (0, 3.2)	8.4 (3.9, 13)	−0.66	0.94	0.6 (0, 2.1)	7.8 (3.4, 12.2)	10.8 (5.8, 15.9)	1.8 (0, 4.1)	0.6 (0, 2.1)	−0.34	1.16
Total	1 (0, 2)	8.7 (5.8, 11.5)	−0.5	1.13	2.2(.06, 3.7	13 (9.7, 16.4)	9.6 (6.7, 12.6)	1.9 (0.5, 3.4)	0.2 (0, 0.8)	−0.55	1.23

*HFA* Height For Age, *BMI* Body Mass Index, *SD* Standard Deviation, *CI* Confidence Interval

**Fig. 2:**
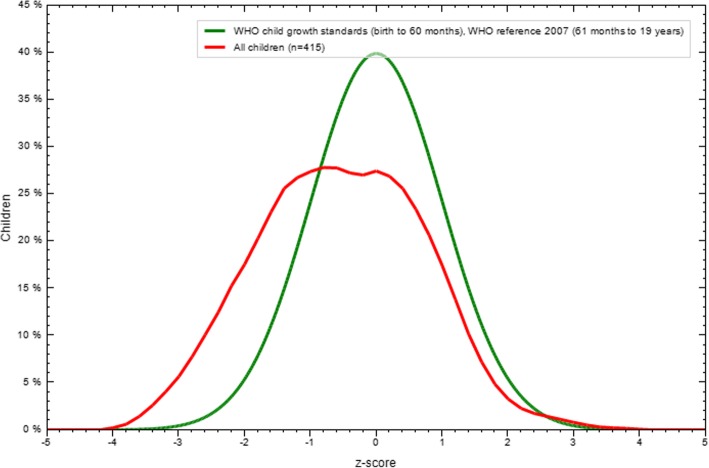
BMI for age result for adolescent refugee girls in Aw – Barre Refugee Camp, Somali Regional state, Southeastern Ethiopia, 2015

The overall wasting (BMI for age) prevalence was 15.2% (95% CI: 11.8, 18.9). Among wasted adolescent girls, 2.2% were severely wasted and 13% were moderately wasted. The overall mean and SD of BMI for age z score was 0.55 ± 1.23. The rate of overweight (> + 1SD) was 11.1%. The rate of wasting among younger adolescents was 19.7% and the magnitude of overweight was 10.8%. On the other hand, the magnitude of wasting and overweight among older adolescents was 8.4 and 13.2% respectively. The distribution of BMI for age Z score under the normal curve as illustrated in Fig. 2 below (Fig. 2).

### Factors associated with stunting and thinness

#### Factors associated with stunting

A crude analysis was done to assess the existence of an association between the independent variables and stunting of the adolescent refugee girls. In the univariate logistic regression; age and duration in the camp were positively associated with the existence of stunting. After fitting all these variables into the multivariable logistic regression, only age was the significant factor. Older adolescent girls were 2 (Adjusted Odds Ratio (AOR): 2.10, 95% CI: 1.12, 3.93) times more likely to develop stunting as compared to younger adolescents as shown in the table below **(**Table 3**).**

**Table 3:** Logistic regression result of associated factors with stunting and thinness among adolescent refugee girls of Aw-Barre Somalia refugee camp, South East Ethiopia, 2015

Logistic regression result for Stunting					
Variables	Category	Stunting		COR (95% CI)	AOR (95% CI)
		Yes	No		
Age	10–14	19	225	1	1
	15–19	26	145	2.12 (1.13, 3.97)	2.10 (1.12, 3.93)*
Duration in the camp (median)	< 8 yrs	5	66	0.58 (0.22, 1.51)	0.60 (0.22, 1.58)
	≥ 8 yrs	40	304	1	1
Logistic regression result for Thinness (Wasting)					
Variables	Category	Wasting		COR (95% CI)	AOR (95% CI)
		Yes	No		
Age in years	10–14	44	200	1	1
	15–19	19	152	0.46 (0.25, 0.84)	1.13 (0.53, 2.40)
Frequency of using protein-rich food sources	used within 2 weeks	4	50	1	1
	used in a month	38	219	2.17 (0.74, 6.35)	1.88 (0.63, 5.59)
	Didn’t use within a month	21	83	3.16 (1.03, 9.75)	2.60 (0.82, 8.18)
Dietary diversity score/DDS	≤ 3 foods items	01	18	0.27 (0.04, 2.03)	0.26 (0.33, 2.02)
	4–5 food items	13	100	0.62 (0.32, 1.19)	0.64 (0.33, 1.24)
	≥ 6 food items	49	234	1	1
Menarcheal status	Post menarche	23	215	0.37 (0.21, 0.63)	0.36 (0.12, 0.75)*
	Pre menarche	40	137	1	1

*Significant at p valve ≤0.05, 1 = reference, *COR* Crude Odd Ratio, *AOR* Adjusted Odd Ratio, *CI* Confidence Interval

#### Factors associated with thinness

A crude analysis was done to assess the existence of an association between the independent variables and thinness of the adolescent refugee girls. In univariate logistic regression; age, starting menstruation, DDS and frequent use of animal products were found to be positively associated variables with the thinness of adolescent girls. After fitting these significant variables into a multivariable logistic regression, only starting menstruation was a significant factor. Adolescents who didn’t start menstruating were 64% (AOR: 0.36, 95%CI (0.12, 0.75)) less likely to be thin/wasted compared to those who did (See in Table 3 Below).

## Discussion

The prevalence of stunting and thinness was 9.7 and 15.2% which is low and moderate public health problem respectively based on this finding. In this study, the magnitude was very low as compared to a study done among Nepal refugees (> 60%) [29] but higher as compared to North Korean refugees [30]. In this study, the magnitude of wasting was comparable with a study done in Wukro, Northern Ethiopia, and the result was 21.6% (95% CI: 15.8, 27.3%). But, the magnitude of stunting was 21.2% (95% CI: 15.8, 27.3%) which is high as compared to our study [31]. This may be due to variation in setting, data collection time, child labor among Nepalese adolescents and duration in the camp with food aid.

Older adolescent girls were 2 (AOR: 2.10, 95% CI (1.12, 3.93) times more likely to develop stunting as compared to younger adolescents. Similarly, it is true among North Korean refugees who were older North Korea children were more likely, and those who lived in South Korea longer were less likely, to be currently stunted [30]. This may be due to the inadequate intake of nutrients during infancy, and childhood strongly affects the linear growth of the recommended height as the respective age. Also, the chronic and cumulative shortage of food, the shortages of basic medicine and fuel, the damage to the infrastructure from wars and the difficult economic circumstances of Somalia might pose substantial challenges to the later life of the adolescent girls’ nutritional status.

Adolescents who didn’t start menstruating were 64% (AOR: 0.36, 95%CI (0.12, 0.75)) less likely to be thin/wasted compared to those who did. Other studies reveal that nutrient deficiency due to menstrual loss, erratic eating habits and preference of foods at the transition age of girls is a common cause for thinness [32, 33]. This may be due to changing habits of food intake because of menstrual-related sign and symptoms and peer influence on food preferences as well as the existence of monotonous family diet due to general ration might be lead to decrement in weight gain which directly affects the BMI for the age of the adolescent girls.

## Conclusion and recommendation

The prevalence of stunting among adolescent refugee girls was a low but thinness or wasting was a moderate public health problem. In this study, the factors associated with the development of stunting were age and for thinness was menarcheal status.

Based on the finding, UNCHR/WFP/ARRA will consider increasing the intake of high caloric food sources with varieties to reduce the prevalence and its intergenerational effect through food fortification, cash transfer and the strengthening of nutrition and public health programs among long-term food aid dependent adolescent refugee girls. Also, the ongoing practice of conducting annual or periodic surveys by stakeholders (UNCHR/WFP/ARRA) among food aid beneficiaries of adolescent girls at least by using anthropometric measurement is important to monitor the prevalence/magnitude.

Health professional should have to provide health and nutrition education and counseling based on age and menarcheal status of adolescent girls by integrating with other reproductive health care services.

## Abbreviations

AOR: Adjusted Odds Ratio
BMI: Body Mass Index
COR: Crude Odds Ratio
DDS: Dietary Diversity Score
HAZ: Height for Age Z score
Hgb: Hemoglobin
SD: Standard Deviation
SPSS: Statistical Packages for Social Sciences
UNHCR: United Nation Higher Commission for Refugee
WAZ: Weight for Age Z score
WHO: World Health Organization
